# Combination Fillings

**Published:** 1892-09

**Authors:** 


ARTICLE V.

### COMBINATION FILLINGS.

The subject of combination fillings is one of great interest to me, and in looking over my records I find that the percentage of these in proportion to the whole number of fillings that I put in is very great. I believe that not one in ten realizes the practical utility of such fillings in the saving of teeth, and I doubt very much i one in twenty uses them to any great extent.

If the teeth are young or old, and of poor quality, to my mind it is much better to put in a combination filling of cement and gold than to fill entirely with gold. Of course, we have all been in the habit of lining up cavities with cements and filling them with gold afterwards. This is a practical and valuable method, and the principle may be extended very materially.

I have been in the habit for some time of putting cement into cavities, and, while the cement is soft, placing plastic gold right into it. By so doing, the gold of itself obtains the necessary retaining points; whereas, if you put the cement in and allow it to harden, then you must re-excavate and form undercuts to hold the gold. In crown cavities, for example, when the central portion of the tooth is largely decayed, but the overlapping enamel is still sufficiently strong to withstand the force of mastication, either amalgam or gold can be put in, and the surplus cement which is squeezed out by forcing the gold or amalgam into the cavity cut away. After waiting a minute or two for the cement to harden, the filling can be furnished with gold or amalgam, as the case may be. It seems to me that in many cases these fillings are better than if they were made of one material. We find oftimes in connection with a large cavity on the mesial surface of a molar, a small pin-head cavity in the distal surface of the adjoining bicuspid. My experience is, that if I fill these smaller cavities with gold, decay will reappear, and the fillings tumble out.

In such cases I often use tin and gold, a combination which you have heard advocated by Dr. Hamilton and Dr. Briggs, and which was spoken of many years ago in an article by Dr. Abbott, of Berlin. It is very servicable in the crown cavities, of sixth-year molars, also in buccal cavities, and in the temporary teeth. The statement which has been made here by Dr. Hamilton and others, that these fillings after a time become hard, something like an amalgam filling, is a fact which I have demonstrated many times in my own practice. One advantage of the combination of tin and gold is that it does not discolor the teeth, as amalgam. As to the use of amalgam and gold, I know that my combination of these materials lasts longer than the all-gold filling which I formerly inserted in similar cases. Oftentimes, in talking with dentists, I ask them if they use this combination, and they will say "yes", and then remark, "Do you use a matrix?" Thus showing that they have no idea of the proper manner of putting in such filling, and consequently, no idea of their value in saving teeth. To correct such false ideas is the reason for my appearing before you to-night.

The use of cement and gold in the front teeth is, to me, a very important affair. Without first putting in cement in the case of large cavities, or moderately large cavities in the poorer quality of teeth, I feel that the chances for permanent success are small. Cavities in frail teeth, where it is difficult to obtain a good retaining shape, can be filled with gold and cement, and the cement will assist very materially in retaining the filling.

In large cavities, if we can have a considerable proportion of the filling cement, we have a better filling, as there is less metal, and the cement will adapt itself better to the tooth than any other material. Here are specimens showing the method of putting in these fillings. This one shows a large approximal cavity in a molar. If a matrix is adjusted and cement placed in the cavity, and, while it is soft amalgam inserted, the cement will be crowded down next to the matrix, and, consequently, the edges of the cavity will not be completely protected by a metal. To overcome this, I put in the cement before putting on the matrix; then add a little amalgam, thoroughly incorporating it with the cement; then trim off around the cavity so that the edge towards the matrix is entirely exposed; then put on the matrix, and finish with amalgam or gold. In other cases I find it convenient to put on the matrix, and put a little amalgam next to it; then fill the body of the cavity with cement and finish with amalgam or gold, or both, as the case may be. The large filling in the lateral incisor, which has been passed around for your inspection, and which, you will remember, is quite large,—restoring the contour of the distal portion of the tooth,—was made in the following manner: The cavity was prepared so that there was considerable undercut at the cervical wall; there was a slight undercut towards the point. Cement was first put in; then plastic gold crowded into the cement; *but the gold did not go into any retaining point whatever.* After waiting a few minutes for the cement to become hard, the filling was finished with odd lots of gold that I picked up around the office, and with an extra hard blow of the automatic mallet. My idea was to see if the blow from the automatic would break up the cement and prevent its being strong. Now, I want this filling tested.* I may suffer a great disappointment to-night, because I don't know how strong the filling is, and it is possible that the gold may have no retention at all in the cement; but I do know that the mallet was put on very much harder than it would have been if the filling had been in the mouth, and the filling was very thoroughly finished with burnishes, plate-files, disks, and stones.

After seeing the pressure it required to break out this filling, I think I should dare trust it in the mouth. Of course, in filling these cavities, you are laboring under unfavorable conditions.—CLAPP, in *Odontographic Journal.*

---

### Editorial, Etc.

*The tooth was then given the President, who removed the filling with strong ezcavators, and stated thst, in his opinion, it was more firmly held in place than would have been possible in such a cavity without the use of cement.

